# Comparative Biochemical and Functional Characterization of a Novel Human Fibrinogen Concentrate (BT524) and Established Plasma-Derived Fibrinogen Concentrates

**DOI:** 10.1055/a-2869-0130

**Published:** 2026-06-24

**Authors:** Michaela Bergmann, Daniela Rübsamen, Steffen Kistner

**Affiliations:** 1 Investigational and Applied BiosciencesBiotest, DreieichGermany

**Keywords:** coagulation/fibrinolysis, biopharmaceuticals, biologics

## Abstract

**Background:**

Human fibrinogen concentrate is used as replacement therapy for congenital and acquired fibrinogen deficiencies. Despite similar manufacturing principles, compositional differences among products may affect clot quality and hemostatic efficacy. BT524 (Biotest) is a newly developed human fibrinogen concentrate with proven clinical safety and efficacy.

**Objectives:**

The objective of this study was to compare the biochemical composition and in vitro functional characteristics of BT524 with those of two commercially available plasma-derived fibrinogen concentrates (Products I and II).

**Methods:**

Three independent batches of each human fibrinogen concentrate were analyzed for the total and clottable protein, fibrinogen antigen, fibrinogen activity by Clauss, clotting factors, degradation markers (fibrinopeptide A and D-dimer), sub-visible particles, and aggregates. Maximum clot firmness and dynamics were assessed using rotational thromboelastometry (fibrin-based thromboelastometry). Fibrinogen deficient plasma with low factor XIII content was spiked each fibrinogen concentrate and increasing concentrations of factor XIII (0–0.8 U/mL).

**Results:**

BT524 showed consistent fibrinogen content but contained no active factor VIII, factor XIII, or von Willebrand factor, indicating a precisely defined composition. Levels of degradation markers, aggregates, and sub-visible particles were markedly lower than in comparator products, reflecting the superior biochemical purity of BT524. BT524 achieved significantly higher maximum clot firmness than Product I ( *p* <0.0001) and demonstrated comparable performance with Product II. Spiking of factor XIII resulted in an additional increase in maximum clot firmness in BT524 and Product II, but not in Product I, proving functional factor XIII susceptibility of BT524.

**Conclusions:**

BT524 demonstrates high biochemical purity and robust functional clot formation. These findings highlight that fibrinogen concentrates differ in composition, functionality and molecular integrity, which determines clot quality and hemostatic performance.

## Introduction

Fibrinogen (coagulation factor I) is an indispensable glycoprotein necessary for clot formation, which promotes platelet aggregation and establishes the fibrin network of clots. Congenital fibrinogen deficiencies such as hypofibrinogenemia and afibrinogenemia alongside acquired fibrinogen deficiency conditions from surgery, postpartum events, liver transplant or trauma hemorrhage markedly elevate bleeding risks. 1​ 2​ ​3​ Replacement therapy with human fibrinogen concentrates (HFCs) is becoming more common due to its standardized fibrinogen content and enhanced pathogen safety along with logistical advantages including quick preparation and administration in small volumes. 1​ 3​​ ​4​ Traditionally, the standard of care for acquired fibrinogen deficiency has been the administration of fresh frozen plasma (FFP) or cryoprecipitate, an approach that remains common practice in the United States and United Kingdom, while several European countries have gradually adopted HFCs as the preferred fibrinogen replacement therapy. 5​

Differences in manufacturing processes, despite similar purification and inactivation steps, can lead to variations in biochemical composition. 6​ These compositional differences may impact crucial aspects of hemostasis, such as clot strength, structure, and susceptibility to lysis. 7​ Therefore, fibrinogen concentrates should not be considered entirely interchangeable, as their distinct *in vitro* properties may lead to differential effects at the clot level. 7​

Fibrinogen concentrate, BT524 (Biotest), is a novel lyophilized product, clinically tested for congenital as well as acquired fibrinogen deficiencies with a proven efficacy and adequate safety and tolerability profile. 8​ 9​ ​10​ In acquired fibrinogen deficiency, BT524 is non-inferior (primary end point) and, in a post-hoc analysis, superior to treatment with FFP or cryoprecipitates (pooled comparator) in reducing intraoperative blood loss during major surgery. Furthermore, it offers practical advantages such as rapid reconstitution and ease of administration. 8​

Fibrinogen concentrates have become more common in medical treatment yet direct comparative studies among diverse plasma-derived products remain scarce. 2​ 6​​ ​11​ The aim of this study was to compare the biochemical composition and *in vitro* functional characteristics of a Biotest fibrinogen concentrate (BT524), with two other commercially available intravenous plasma-derived HFCs (referred to as Product I and Product II). This study will help to understand how product-specific attributes may influence hemostatic efficacy, thereby contributing to more tailored fibrinogen replacement therapies in patients with bleeding disorders.

## Materials and methods

### Fibrinogen reconstitution

Three different batches for each comparator product (Product I and Product II) were obtained commercially. Fibrinogen concentrates are provided as lyophilizates, and they were reconstituted according to the product-specific package insert, i.e., 1 g per vial reconstituted in 50 mL water for injection, therefore resulting in solutions with a nominal value of 20 mg/mL fibrinogen. After successful reconstitution, which was verified via visual inspection, all products were filtered through an enclosed filter system or a 17 µm Pall filter (for Product II).

Fibrinogen (Clauss), clottable protein, sub-visible particles, fragments/aggregates, factor VIII (FVIII), factor XIII (FXIII), and von Willebrand factor (vWF) activity were measured in freshly reconstituted samples. For the remaining assays, aliquots were prepared and stored at≤− 70°C. Repeated freeze–thaw cycles were avoided.

### Fibrinogen content and activity assays

The amount of clottable fibrinogen was determined using a plate-based assay where the total protein value was measured via optical density. Afterwards, the active clot-forming fibrinogen was activated by adding calcium chloride and an excess of thrombin at 37°C in imidazole buffer, pH 6.7, for 20 minutes at 37°C. 12​ Non-clotted components of the sample remained in the supernatant, and the total protein content thereof was measured via optical density. The clottable protein was calculated by subtraction of the remaining protein in the supernatant from the total protein. The specific fibrinogen activity was calculated by dividing the clottable protein by the total protein after albumin subtraction. No albumin was subtracted for BT524 as, in this product, the albumin content was below the detection limit.

Fibrinogen antigen was determined by a standard nephelometric assay using a BN ProSpec analyzer using the respective antisera and standard human serum as calibrators (Siemens).

Fibrinogen activity was measured by Clauss methods using a coagulation analyzer (BCS XP) by adding a large excess of thrombin (Multibrin U, Siemens) and measuring the coagulation time. Fibrinogen concentration was calculated using a fibrinogen calibrator kit (Siemens).

The maximum clot firmness (MCF) was determined with rotational thromboelastometry (ROTEM delta, Werfen). The MCF of the fibrin clot using the fibrin-based thromboelastometry (FIBTEM) method, including the addition of cytochalasin D (FIBTEM reagent), was measured in fibrinogen-deficient plasma spiked with the respective HFCs. Three different fibrinogen concentrations (1.0, 2.5, and 3.5 mg/mL) were spiked, based on the nominal fibrinogen values of each concentrate (20 mg/mL for all products), and tested in the assay.

### Measurement of clotting factors

Coagulation factor activities for factors II, VII, VIII, IX, X, XI, and XII were determined using a coagulation analyzer (BCS XP) with standard one-stage clotting assays using Siemens reagents in their respective deficient plasmas and standard human plasma as calibrators while factor XIII was dertemined by the Berichrom FXIII kit by Siemens.

vWF activity was quantified using a coagulation analyzer (BCS XP) by a particle-enhanced immunological turbidity test using the Innovance test kits. A turbidity test with the BCS XP was used to assess the vWF antigen.

### Measurement of degradation products

Fibrinopeptide A (FPA) was determined with the commercially available competitive FPA from Hyphen BioMed using a plate reader (POLARstar Omega). Before execution of the enzyme-linked immunosorbent assay, fibrinogen was removed from the samples through centrifugation with Vivaspin 50 kDa columns and bentonite treatment.

D-Dimer was analyzed using a coagulation analyzer (BCS XP) by a particle-enhanced immunological turbidity test using the Innovance test kit.

Sub-visible particles were quantified, without prior filtration of the products using a particle counter (HIAC 9703+). Particles≥10 µm and ≥ 25 µm were distinguished and reported as counts per measurement, which included four measurements in 100 mL of sample volume.

Aggregates and fragments of fibrinogen molecules were determined using high performance size-exclusion chromatography (HPSEC). The peak that eluted shortly before the fibrinogen monomer peak was assigned as the aggregate peak. The small peaks after the fibrinogen monomer were assigned to fragments. The percentage of the respective peak area was reported.

### Clot firmness dynamics

Fibrinogen-deficient plasma with low FXIII content (15.3% of norm) was spiked with 2.5 mg/mL of fibrinogen concentrates of the different products and increasing concentrations of FXIII (Fibrogammin, CSL): 0, 0.2, 0.4, 0.6 and 0.8 U/mL. Hemostasis was assessed by the FIBTEM method (ROTEM, Werfen) and the following parameters were analyzed: MCF, clot formation time (CFT), amplitude 5 minutes after clotting (A5) and amplitude 10 minutes after clotting (A10).

### Supplementary methods

Fibronectin, albumin and α2-macroglobulin were determined by standard nephelometric assays using a nephelometric analyzer (BN ProSpec) using the respective antisera and standard human serum as the calibrator (Siemens).

Proteolytic activity was determined using a coagulation analyzer (BCS XP) by measuring the release of p-nitroaniline (pNA) from the model peptide S-2288 (H-D-Ile-Pro-Arg-pNA) by absorbance at 405 nm.

The non-activated partial thromboplastin time (NaPTT) was performed using a coagulation analyzer (BCS XP) based on Ph. Eur. 2.6.22 using a 1:4 instead of 1:10 and 1:100 sample dilution to increase sensitivity. NaPTT values were reported as coagulation time in seconds and the coagulation time normalized to the value of the test buffer (negative control) in percent.

Concentrations of immunoglobulins (IgG, IgA, and IgM) were determined using the analyzer Alinity c from Abbott. The calibration was performed in the lower assay range to increase sensitivity.

Structural integrity of fibrinogen was analyzed by sodium dodecyl sulfate–polyacrylamide gel electrophoresis (SDS-PAGE) and Western blotting. For SDS-PAGE, 2 µg of nominal fibrinogen per sample were separated under reducing conditions on 4–12% bis–tris gels and visualized by Coomassie staining. Gels were recorded using an Odyssey imaging system (LI-COR Biosciences).

For Western blot analysis, 0.4 µg of nominal fibrinogen per sample were separated by SDS-PAGE and probed with antibodies against the fibrinogen α, β and γ chains (mouse anti-human fibrinogen α, Santa Cruz Biotechnology; goat anti-human fibrinogen β and rabbit anti-human fibrinogen γ, both Biorbyt). Appropriate fluorescent secondary antibodies were used for detection, and signals were recorded using a Typhoon scanner (Cytiva).

### Statistical analysis

To describe the variability of each measured parameter, the mean value and the standard deviation were reported. Graphs and statistical evaluations were conducted using GraphPad Prism version 10.2.3. One-way analysis of variance (ANOVA) was performed to compare BT524 and other fibrinogen products, followed by Dunnett’s multiple comparisons test. Two-way ANOVA between BT524 and products was conducted with Šídák's multiple comparisons test for post hoc analysis. Adjusted *p* -values were reported as follows: *p* <0.05 (*), *p* <0.01 (**), *p* <0.001 (***), *p* <0.0001(****), and not significant (ns) for *p* ≥0.05. The respective statistical tests applied were indicated in the figure legends.

## Results

We compared the biochemical characteristics of BT524 against three batches each of two commercially available fibrinogen concentrates, hereafter referred to as Products I and II.

### Fibrinogen content and activity

**Fig. 1:**
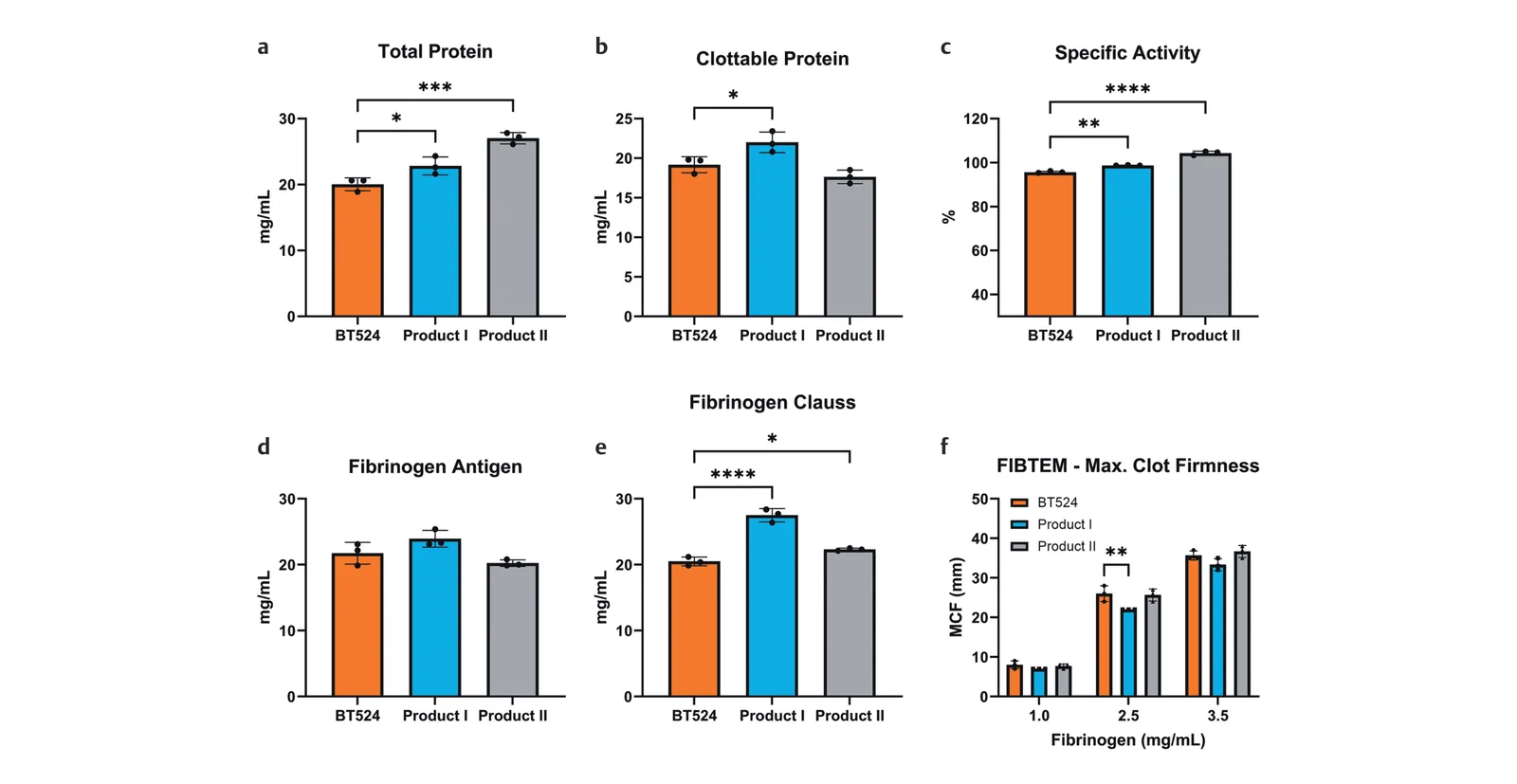
Fibrinogen content and activity of BT524, Product I and Product II. Total protein ( **a** ) and clottable protein ( **b** ) were measured via a plate-based method with optical density; specific activity ( **c** ) was calculated by clottable protein/(total protein–albumin); fibrinogen antigen ( **d** ) and Clauss ( **e** ) were measured via a coagulation analyzer and maximum clot firmness (MCF) ( **f** ) was measured via thromboelastography. Tests were performed in three batches each. One-way ANOVA was performed with *p* <0.05 (*), *p* <0.01 (**), *p* <0.001 (***) and *p* <0.0001 (****).

All fibrinogen concentrates were formulated to achieve a final nominal concentration of 20 mg/mL clottable (active) fibrinogen upon reconstitution. For BT524, the total protein was 20.04±0.82 mg/mL, clottable protein was 19.16±0.83 mg/mL, fibrinogen antigen was 21.72±1.33 mg/mL and fibrinogen activity by Clauss was 20.50±0.54 mg/mL. These results yielded a specific activity of 95.62±0.34% ( Fig 1 ).

Product I exhibited total protein (22.85±1.10 mg/mL), clottable protein (22.02±1.06 mg/mL) and activity measured by Clauss (27.50±0.83 mg/mL), i.e. significantly higher compared with BT524; whereas the fibrinogen antigen concentration (23.92±1.05 mg/mL) was found to have a similar level to BT524. This resulted in a higher specific activity (98.67±0.12%). In contrast to the data reported for fibrinogen activity, Product I exhibited lower MCF values (21.83±0.24 mm) when samples were spiked with 2.5 mg/mL fibrinogen, compared with BT524 (25.67±1.65 mm). This might indicate a mechanically less robust clot formed after the administration of Product I ( Fig 1 ).

Product II showed slightly lower values for clottable protein (17.64±0.68 mg/mL) and fibrinogen antigen (20.18±0.40 mg/mL) compared with BT524. Fibrinogen activity (Clauss; 22.30±0.16 mg/mL) was slightly higher than BT524. Likewise, the total protein (27.04±0.68 mg/mL) was significantly higher than BT524 ( Fig 1 ), mainly due to the high albumin content of 10.13±0.23 mg/mL ( **Supplementary Table 1** , available in the online version only) in the product. Consequently, an overall high specific activity over 100% (104.29±0.71%) was calculated for Product II.

### Other clotting factors

**Fig. 2:**
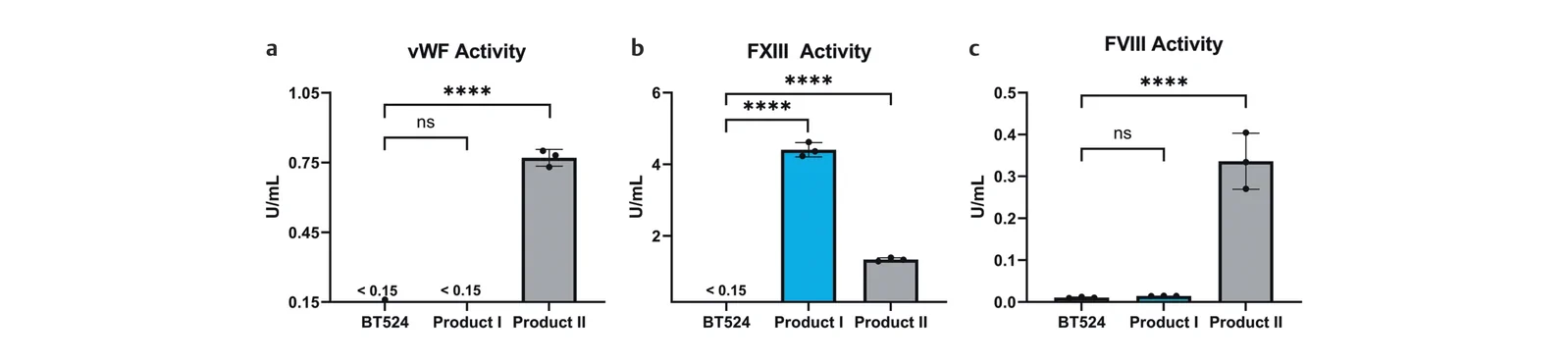
Von Willebrand factor (vWF) ( **a** ), FXIII ( **b** ) and FVIII ( **c** ) activities among three fibrinogen concentrates: BT524, Product I and Product II. Tests were performed in three batches each at a coagulation analyzer (vWF via a turbidity test, FVIII via a clotting assay and FXIII via a chromogenic assay). vWF activity led in two of three batches of BT524 to values below the detection limit and for one batch a value could be generated (depicted as a black dot in the graph). One-way ANOVA was performed with *p* <0.0001 (****) and not significant (ns) for *p* ≥0.05 *.*

Clotting factors in all three fibrinogen concentrates were below the detection limit for FII, FVII, FIX, FX and FXI. vWF antigen levels were higher in BT524 (3.05±0.16 U/mL) and Product II (3.39±0.37 U/mL) than in Product I (0.19±0.02 U/mL; **Supplementary Table 1** , available in the online version only). Remarkably, in BT524 and Product I, none of the vWF was active (with only one of three batches above the detection limit of 0.15 U/mL in the Biotest product). Conversely, Product II exhibited a measurable vWF activity of 0.77±0.03 U/mL, which was significantly higher than BT524 ( *p* <0.0001; Fig 2a ).

FXIII activity was significantly higher in Product I (4.40±0.16 U/mL, *p* <0.0001) and in Product II (1.33±0.04 U/mL, *p* <0.0001) compared with BT524, which was below the detection limit of 0.15 U/mL ( Fig. 2b ).

FVIII activity was very low for BT524 and Product I (0.01±0.00 U/mL), whereas for Product II, it was significantly higher (0.34±0.05 U/mL, *p* <0.0001; Fig 2c ).

### Degradation products, aggregates and particles

**Fig. 3:**
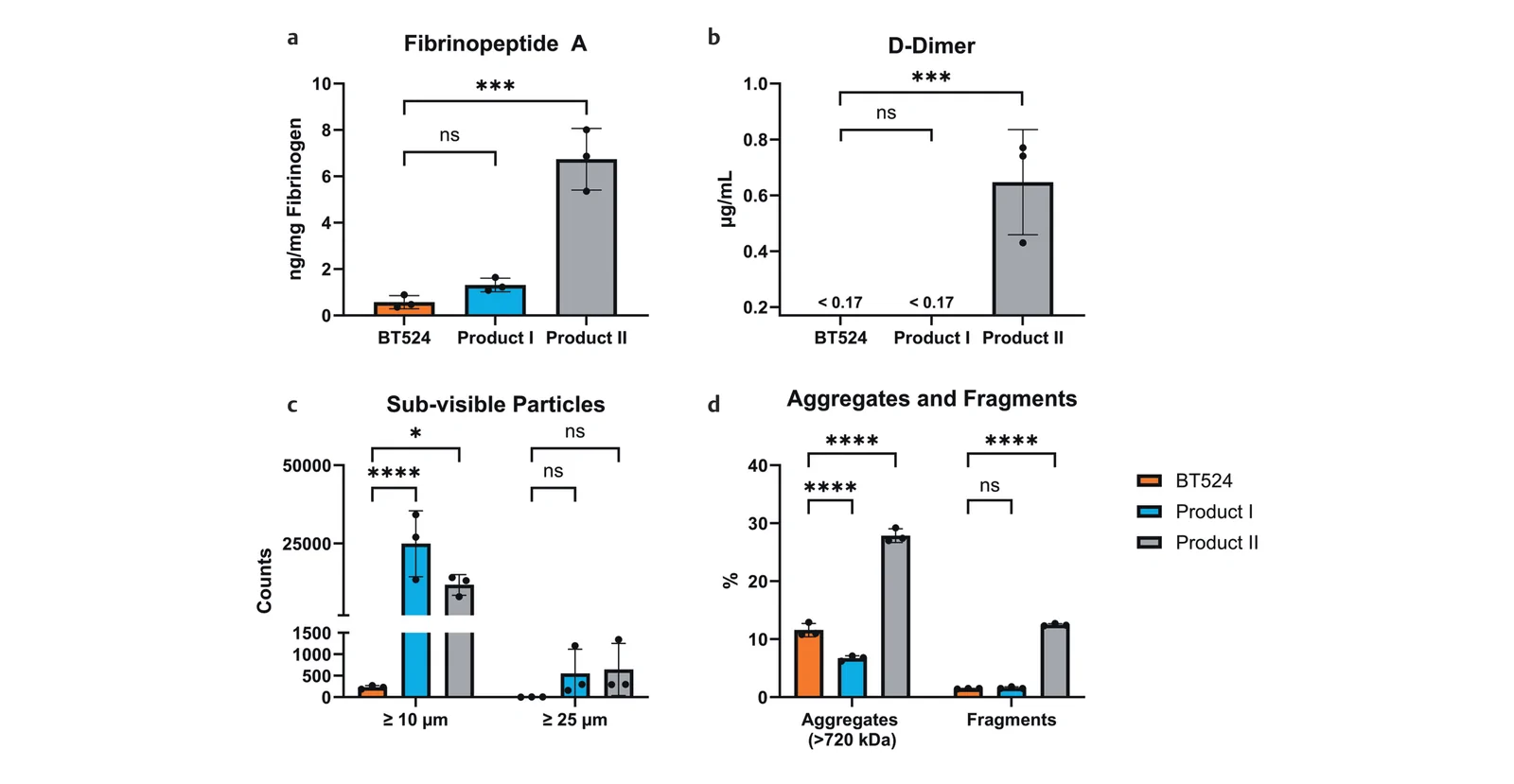
Degradation products of BT524 and two comparator products (Product I and Product II): fibrinopeptide A (FPA) ( **a** ) and D-Dimer ( **b** ) were measured via the ELISA and turbidity test, respectively. Sub-visible particles ( **c** ) were measured using a particle counter distinguishing between≥10 µm and≥25 µm particles sizes. Percentage of aggregates or fragments ( **d** ) were detected via HPSEC. Tests were performed in three batches each. One-way ANOVA was performed with *p* <0.05 (*), *p* <0.001 (***), *p* <0.0001 (****), and not significant (ns) for *p* ≥0.05.

We further tested for the degradation products FPA and D-Dimer ( Fig. 3a and b ). Since FPA is released by fibrinogen during the cleavage and activation by thrombin, it serves as a surrogate marker for unwanted activation during the fibrinogen production process. We found a low FPA content in the BT524 product (11.43±4.67 ng/mL, corresponds to 0.57±0.23 ng/mL fibrinogen), indicating no unwanted activation. Conversely, FPA levels were slightly elevated, although not statistically significant, in product I (26.28±4.76 ng/mL, corresponds to 1.31±0.24 ng/mL fibrinogen), whereas the increase was statistically significant in Product II (134.75±21.76 ng/mL, corresponds to 6.74±1.09 ng/mL fibrinogen; *p* <0.0001). A similar pattern was observed for D-Dimer, which was below the detection limit for BT524 and Product I, but strongly elevated in Product II (0.65±0.15 µg/mL, *p* =0.0006), indicating signs of fibrinolysis or co-purification of cleaved fibrin.

The other HFC products contained significantly higher numbers of sub-visible particles≥10 µm (Product I: 24920.33±8599.57, *p* <0.0001; Product II: 11764.33±2684.41, *p* 0.0161) than BT524 (231.00±35.14; Fig. 3c ). These measurements were conducted before filtration of the samples, thereby reflecting differences in the manufacturing processes rather than patient safety. Also, the number of sub-visible particles≥25 µm was slightly higher for the comparator products than for BT524 without statistical significance.

Aggregates exceeding 720 kDa (corresponding to complexes of more than two fibrinogen molecules bound together) were present at lower levels in Product I (6.70±0.37%, *p* <0.0001) than in the BT524 product (11.57±0.95%). In contrast, Product II (27.87±0.96%, *p* <0.0001) exhibited a significantly higher proportion of aggregates ( Fig 3d **;** representative chromatograms of one batch for each product are shown in **Supplementary Fig. 1** , available in the online version only). In line with the D-Dimer data, we found significantly more fragments in Product II (12.47 ± 0.17%, *p* <0.0001), than in BT524 (1.47±0.05%) or Product I (1.60±0.14%). These findings were supported by SDS-PAGE and Western blot analysis ( **Supplementary Fig. 2** , available in the online version only), which confirmed the presence of intact fibrinogen chains while reflecting the higher abundance of aggregated species in Product II compared with BT524 and Product I.

### Clot firmness dynamics

**Fig. 4:**
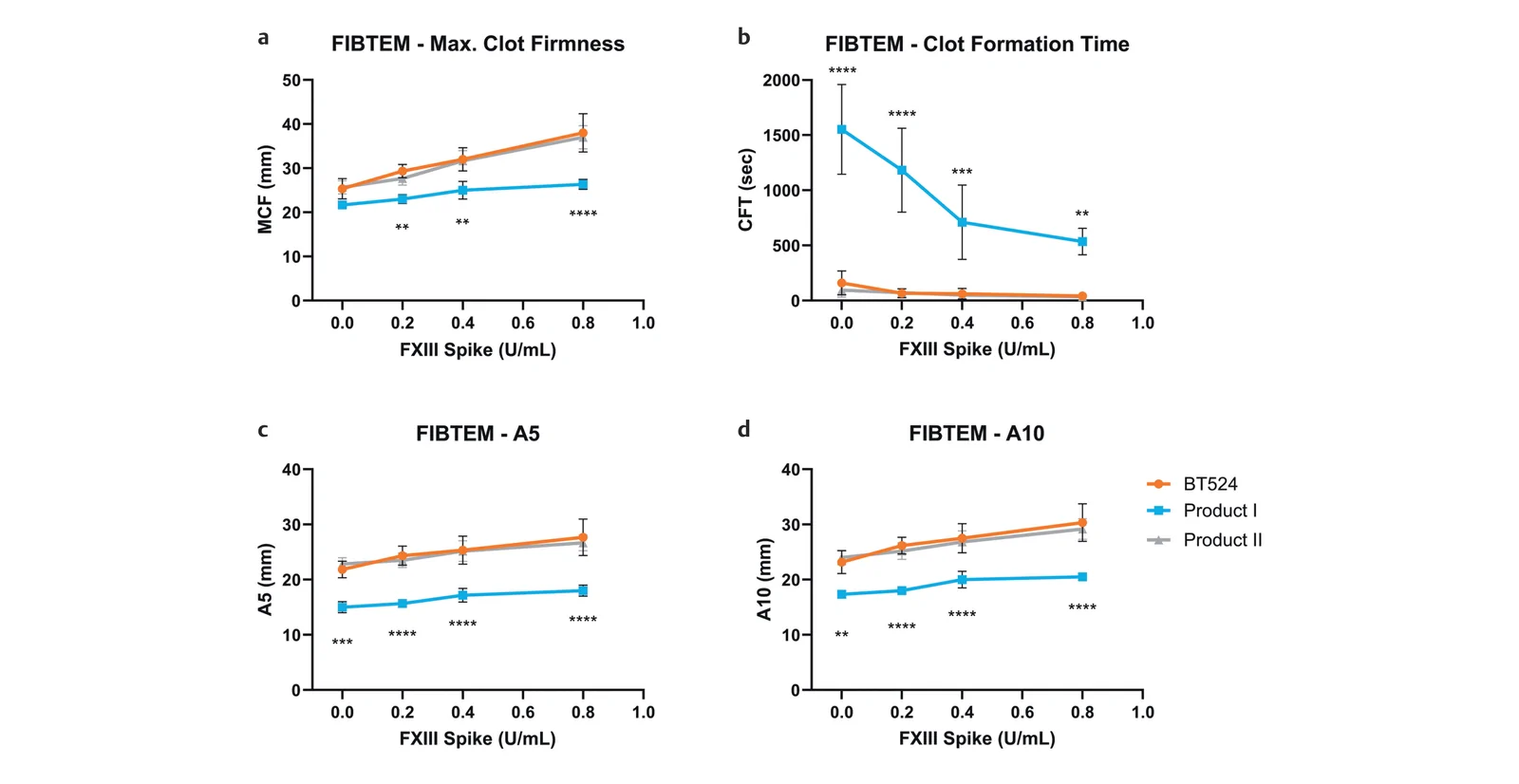
Clot firmness dynamics of BT524 and two comparator products (Product I and Product II): Maximum clot firmness (MCF) ( **a** ), clot formation time (CFT) ( **b** ) and amplitude 5 minutes ( **c** ) and 10 minutes ( **d** ) after clotting were measured with different FXIII spikes in 2.5 mg/mL of the three fibrinogen products in fibrinogen-deficient plasma via FIBTEM measurements at ROTEM. Tests were performed in three batches each. Significance was determined via two-way ANOVA with *p* <0.01 (**), *p* <0.001 (***), *p* <0.0001 (****).

To investigate the potential functional impact of the fibrinogen concentrates, FIBTEM measurements were performed in samples spiked with FXIII (see Material and methods/Clot firmness dynamics) in order to evaluate the CFT, the MCF, the A5 and the A10. As shown above, Product I was found to already contain 4.40 U/mL FXIII as part of the product. Nonetheless, we spiked the same quantity of FXIII into all products, independent of their native FXIII content. We observed a correlation for BT524 and Product II, where increasing amounts of FXIII led to a stronger clot, measured by increased clot firmness. However, for Product I, the clot firmness was significantly lower than for BT524 ( *p* <0.0001 at 0.8 U/mL FXIII spike) and Product II, reaching a saturation at about 0.4 U/mL additional FXIII ( Fig. 4a ).

A similar effect was seen for the CFT: even the addition of 0.8 U/mL FXIII could not decrease the CFT to the same level as in BT524 or Product II ( Fig. 4b ). In line with the MCF and the CFT, also the A5 and A10 after FXIII spikes showed a significantly weaker clot with Product I compared with BT524 ( *p* <0.0001), while Product II was similar to BT524 ( Fig. 4b–c ).

Taken together, while BT524 showed similar fibrinogen content and activity to those of the comparator products, it contains less active coagulation factors, degradation products, sub-visible particles and fragments than the comparator Product II. Furthermore, BT524 shows functional superiority in comparison with Product I when considering clot firmness and CFT.

## Discussion

The newly developed HFC BT524 offers a highly defined and biochemically pure composition containing active and polymerization-competent fibrinogen at the specified concentration. It is essentially free from accompanying proteins or degradation products. Quantitative characterization by clottable and antigen assays revealed a plausible, consistent high specific activity across all tested lots, contributing to functional clot formation.

A particularly notable result of this study is a significantly higher MCF of BT524 compared with Product I, although values for total protein, clottable protein, specific activity and fibrinogen Clauss were slightly lower. Total or clottable protein measurements represent compositional parameters, 12​ and the Clauss assay primarily reflects thrombin responsiveness, 13​ whereas viscoelastic methods such as FIBTEM provide a functional assessment. FIBTEM captures the kinetics and quality of fibrin network formation under dynamic conditions that mimic physiological settings more closely than endpoint-based assays. It reflects not only the presence of thrombin-cleavable fibrinogen, but also its polymerization competence and structural integrity, both of which are critical for mechanical clot formation, therefore, providing a global evaluation of hemostasis. 14​

It is frequently observed that the Clauss assay yields higher values than clottable protein measurements, as the Clauss method also detects fibrinogen aggregates that remain thrombin-reactive but are not necessarily capable of forming functional fibrin networks. 15​ This is in line with the fibrinogen levels measured by Clauss in Product II, which exhibited statistically significantly higher fibrinogen levels and also showed a markedly increased number of fibrinogen aggregates. Although Product I does not contain higher amounts of aggregates, it shows strongly elevated sub-visible particles (≥10 µm), which could also influence fibrinogen determination by the Clauss method, as optical clot detection is known to be affected by sample turbidity. 16​ These factors likely contributed to elevated fibrinogen Clauss values, while clottable protein levels remained comparable with BT524. The observation that Product II exhibited a specific activity theoretically exceeding 100% is possibly related to the albumin content of the product. Albumin may lead to an underestimation of the total protein content determined via optical density due to its different molar extinction factor compared with fibrinogen. Moreover, the artificially high fibrinogen Clauss value of the comparator products may complicate accurate dosing decisions in patients. 14​

Characterization of BT524 demonstrated the absence of clotting factors (e.g., FVIII, FXIII) and albumin, and only minimal traces of the vWF antigen were detectable. Importantly, no vWF activity was measured, indicating that the trace vWF antigen likely represented a biologically inactive form. This level of molecular cleanliness ensures that the observed functional clot behavior is attributable solely to the fibrinogen molecule itself. Even prior to a filtration step, BT524 was already almost free of sub-visible particles, thereby minimizing the potential risk of immunogenicity of the product. 17​

Despite containing neither FXIII nor excess fibrinogen, the new HFC BT524 consistently achieved superior clot firmness compared with Product I in ROTEM-based FIBTEM assays. Product I, which contained both higher fibrinogen clottable protein and elevated endogenous FXIII activity, showed markedly inferior performance in all FIBTEM parameters measured. Supplementation with increasing concentrations of exogenous FXIII led only to slight increases in clot formation performance for Product I, significantly lower than that for BT524. This suggests that cross-linking potential alone cannot compensate for reduced polymerization competence imposed by molecular fragmentation or structural impairment. These results align with the literature on dilutional coagulopathy, where FXIII supplementation enhances MCF when combined with fibrinogen, but its effect remains limited or absent when the fibrinogen substrate is structurally compromised. 18​ When fibrinogen is degraded or conformationally altered, polymerization is not efficient, weaker fibrin networks are formed, and its susceptibility to fibrinolysis is increased. 18​ ​19​

This study was conducted entirely under in vitro conditions and therefore cannot fully replicate the complex, dynamic physiological environment of hemostasis in vivo. In vitro assays such as ROTEM and biochemical analyses provide controlled insights into fibrin polymerization and clot mechanics, but they do not capture the influence of vascular endothelium, blood flow dynamics, platelet activation, or cellular interactions. 20​ Moreover, only a limited number of batches and commercially available fibrinogen concentrates were examined, which may not represent the full manufacturing variability across production scales or lots.

In summary, the in vitro properties observed for BT524 appear to be primarily influenced by the structural integrity, compositional homogeneity, and biochemical characteristics of its fibrinogen component. These findings may provide valuable insights into its mechanism of action and enhance the broader understanding of its potential clinical relevance.

## Supplementary Material

Supplementary Material Zusatzmaterial
