# Supplementary material for: Comparative Biochemical and Functional Characterization of a Novel Human Fibrinogen Concentrate (BT524) and Established Plasma-Derived Fibrinogen Concentrates
Source: Drug Res (Stuttg). 2026 Jun 24;76(7):179–85. doi: 10.1055/a-2869-0130 (PMC13588683; doi:10.1055/a-2869-0130)
Supplement: Supplementary file 1 [file 10-1055-a-2869-0130_29440955.pdf]

Supp. Table 1. Summary of biochemical and functional parameters measured for BT524, Product I, and Product II.

Parameter		BT524 (n=3)	Product I (n=3)	Product II (n=3)
**Aggregates (> 720 kDa)**	%	11.57 ± 0.95	6.70 ± 0.37	27.87 ± 0.96
**Albumin**	mg/mL	< 0.044 ± 0.00	0.54 ± 0.06	10.13 ± 0.23
**α_2-macroglobulin**	mg/mL	< 0.046 ± 0.00	0.07 ± 0.00	0.62 ± 0.22
**D-Dimer**	µg/mL	< 0.17 ± 0.00	< 0.17 ± 0.00	0.65 ± 0.15
**FII**	U/mL	< 0.05 ± 0.00	< 0.05 ± 0.00	< 0.05 ± 0.00
**FVII**	U/mL	< 0.10 ± 0.00	< 0.10 ± 0.00	< 0.10 ± 0.00
**FVIII**	U/mL	0.01 ± 0.00	0.01 ± 0.00	0.34 ± 0.05
**FIX**	U/mL	< 0.05 ± 0.00	< 0.05 ± 0.00	< 0.05 ± 0.00
**FX**	U/mL	< 0.05 ± 0.00	< 0.05 ± 0.00	< 0.05 ± 0.00
**FXI**	U/mL	< 0.02 ± 0.00	< 0.02 ± 0.00	< 0.02 ± 0.00
**FXII**	U/mL	< 0.01 ± 0.00	0.04 ± 0.00	< 0.01 ± 0.00
**FXIII**	U/mL	< 0.15 ± 0.00	4.40 ± 0.16	1.33 ± 0.04
**Fibrinogen**				
**Clauss method**	mg/mL	20.50 ± 0.54	27.50 ± 0.83	22.30 ± 0.16
**Clottable protein (total protein – non-clotted protein)***	mg/mL	19.16 ± 0.83	22.02 ± 1.06	17.64 ± 0.68
**Antigen**	mg/mL	21.72 ± 1.33	23.92 ± 1.05	20.18 ± 0.40
**Fibrinopeptide A**	ng/mL	11.43 ± 4.67	26.28 ± 4.76	134.75 ± 21.76
**Fibrinopeptide A**	ng/mg Fibrinogen	0.57 ± 0.23	1.31 ± 0.24	6.74 ± 1.09
**Fibronectin**	mg/mL	0.23 ± 0.04	0.16 ± 0.04	3.85 ± 0.72
**FIBTEM - MCF at 2.5 mg/mL Fibrinogen**	mm	25.67 ± 1.65	21.83 ± 0.24	25.50 ± 1.08
**Fragments**	%	1.47 ± 0.05	1.60 ± 0.14	12.47 ± 0.17
**IgA**	mg/mL	< 0.03 ± 0.00	< 0.03 ± 0.00	< 0.03 ± 0.00
**IgG**	mg/mL	< 0.3 ± 0.00	< 0.3 ± 0.00	< 0.3 ± 0.00
**IgM**	mg/mL	< 0.3 ± 0.00	< 0.3 ± 0.00	< 0.3 ± 0.00
**NaPTT**	%	124.00 ± 1.63	126.00 ± 1.63	125.00 ± 3.56
**Proteolytic activity**	U/L	< 3.0 ± 0.00	< 3.0 ± 0.00	< 3.0 ± 0.00
**Specific Activity [clottable protein / (total protein - albumin)]***	%	95.62 ± 0.34	98.67 ± 0.12	104.29 ± 0.71
**Sub-visible particles**				
**≥ 10 µm**	Counts	231.00 ± 35.14	24920.33 ± 8599.57	11764.33 ± 2684.41
**≥ 25 µm**	Counts	4.67 ± 1.25	553.33 ± 460.82	646.67 ± 495.22
**Total protein**	mg/mL	20.04 ± 0.82	22.85 ± 1.10	27.04 ± 0.68
**vWF activity**	U/mL	0.16 (n = 1 of 3); < 0. 15 ± 0.00 (n=2 of 3)	< 0.15 ± 0.00	0.77 ± 0.03
**vWF antigen**	U/mL	3.05 ± 0.16	0.19 ± 0.02	3.39 ± 0.37

*see Materials and methods for detailed description

Drug Res | DOI 10.1055/a-2869-0130 | © The Author(s).
